# Surveying the oral health needs of international students in Canada

**DOI:** 10.3389/froh.2026.1766729

**Published:** 2026-04-22

**Authors:** Bianca Jamal, Maria Khan, Hassan W. Yassin, Shahzaib Fida, Jessica Lieffers, Amrinderbir Singh

**Affiliations:** 1 College of Dentistry, University of Saskatchewan, Saskatoon, SK, Canada; 2 College of Pharmacy and Nutrition, University of Saskatchewan, Saskatoon, SK, Canada

**Keywords:** access to care, dental health survey(s), dental public health, nutrition/nutritional sciences, preventative dentistry, social determinants

## Abstract

**Objectives:**

Oral health plays a significant role in overall health, influencing essential functions of eating, breathing, speaking, and can have psychosocial impacts. Research from various countries indicates that international students face unique barriers to maintaining optimum oral health, shaped by financial, systemic, and cultural factors; however, limited Canadian research exists on this topic. This study explored the oral health knowledge, attitudes, and practices of international students at the University of Saskatchewan (USask).

**Methods:**

A 75-item survey instrument gathered data from USask international students. The survey explored oral health knowledge, attitudes, and preventive practices. Quantitative data were analyzed using SPSS 28.0.1.0; qualitative data were analyzed through thematic coding using Excel.

**Results:**

In total, *n* = 56 international students were included in the analysis. While 98.0% (*n* = 50) of respondents considered their oral health important, 40.4% (*n* = 21) felt they did not have adequate knowledge to access oral care. In total, 55.4% (*n* = 31) of respondents brushed twice daily. Overall, 32.1% (*n* = 21) of international students rated their oral health as “fair” or “poor”. Notably, 40.0% (*n* = 22) of respondents reported having untreated mouth problems. In terms of diet, about half of respondents reported consuming more sugary foods (52.0%; *n* = 26) and sugary drinks (50.0%; *n* = 25) in Canada compared to when they were in their home country.

**Conclusion:**

This study's findings suggest that there are some oral health concerns in this group. Expanding this research nationally across universities and including domestic students for comparison would strengthen an understanding of international students’ oral health and inform future interventions that could help address these oral health inequities.

## 1 Introduction

Oral health is a significant, yet often underappreciated aspect of overall health. It plays a central role in performing essential functions such as eating, breathing, and speaking, while also impacting psychosocial aspects including mental health, self-esteem, and confidence (1, 2). Moreover, the oral cavity is the intersection of medicine and dentistry, functioning as the primary gateway to the rest of our body (3). It is an important indicator of systemic conditions such as diabetes, atherosclerotic vascular disease, pulmonary disease, pregnancy-related complications, osteoporosis, and kidney disease, among others (3). Accordingly, oral health must be a core component of general health—deserving similar attention and investments as systemic health.

Access to oral healthcare, however, is far from equitable. Vulnerable populations in Canada, including refugees and immigrants, children, seniors in long term care, Indigenous communities, individuals with disabilities, and low-income families, face significant barriers to maintaining good oral health (4). These systemic barriers include social, cultural, economic, and geographic issues (5).

Among those navigating oral health inequities in Canada are international students. According to the Government of Canada, an estimated 997,820 international students had valid study permits as of December 31, 2024 (6); these numbers have also been rising over the past decade (5). International students originate from across the globe, bringing unique cultural values, health beliefs, diets, and expectations regarding healthcare. However, the current healthcare system may not fully align with addressing their oral healthcare needs, particularly when providing culturally appropriate and accessible care (7).

A recent scoping review of international students from various home and host countries found that international students generally experience worse oral health outcomes compared to their domestic peers (7) including higher rates of decayed, missing, and filled teeth (DMFT), increased calculus accumulation, and poorer periodontal health and oral hygiene-related issues (8–11). Numerous studies that captured mainly self-reported data also reported infrequent or no dental visits (8, 12–18). Contributing factors to worse oral health outcomes included language barriers, culture shock, financial constraints, mental health challenges, and lack of familiarity with the healthcare system (8, 19). However, none of the studies identified Canada as a host country, and the few available Canadian national health surveys that do capture oral health data do not identify international students, making it difficult to ascertain the oral health status of this group in this country. Given the general impact of poor oral health on university students’ academic success (20), mental well-being (21), and social engagement (22), it is necessary to better understand and address these challenges encountered by international students.

Further, studies have often overlooked the intersectionality and complex determinants of oral health among international studies, including known links between nutrition and oral health (23–25), such as dietary changes, food insecurity, and limited access to nutritious foods (8).

Since many international students stay in Canada post-graduation, contributing to the workforce and broader society (26), it is important that they are equipped with the knowledge, resources, and access to maintain good oral health as a matter of health equity and long-term public interest.

The lack of targeted, comprehensive Canadian data on oral health in international students hinders post-secondary institutions from providing inclusive oral health promotion resources and policies. Thus, this project aimed to explore the self-reported knowledge, attitudes, and practices regarding oral health, oral health preventative and therapeutic practices, and diet of international students in Saskatchewan, Canada.

## 2 Methods

### 2.1 Study design

A cross-sectional survey methodology was employed, with study protocol informed by a recent scoping review identifying limited and inconsistent data on international students’ oral health, particularly in Canadian contexts (7).

### 2.2 Participants and recruitment

International students enrolled at the University of Saskatchewan (USask) were eligible to participate. Recruitment was conducted in collaboration with the international student centre and student associations, using various channels, including print and digital posters, internal university communications, social media posts, word of mouth, and electronic communication newsletters. The survey response period was from February 23, 2025 to June 21, 2025.

### 2.3 Survey instrument

Self-reported data were gathered through an anonymous online survey administered through Survey Monkey. The questionnaire was adapted from the oral health component of Statistics Canada's Canadian Oral Health Survey Cycle 1 (2023–2024) (27) and the Canadian Health Measures Survey Cycle 1 (2007–2009) (35), with additional questions based on relevant literature and a recent scoping review on international students’ oral health (7). The survey included 75 questions, including 66 closed-ended questions (Likert scale, dichotomous, multiple choice) and nine open-ended questions.

The survey's content validity was assessed by a panel of expert reviewers from the University of Saskatchewan Student Union (USSU) and International Student and Study Abroad Centre (ISSAC). Further, cognitive interviews were conducted by AS with two volunteer USask international students; they verbalized their inner monologue while completing the draft survey and research team members took detailed notes (28, 29). Feedback from these survey development strategies resulted in revisions to improve clarity, cultural relevance, and validity prior to survey administration.

### 2.4 Key variables

The key independent variables are shown in Supplementary Table S1, such as degree pursued, academic unit, and age. Supplementary Table S2 shows key dependent variables such as measures of oral health knowledge, attitudes, and practices including diet.

### 2.5 Quantitative data analysis

Closed-ended question survey responses underwent quantitative analysis using SPSS 28.0.1.0. Descriptive statistics were used to summarize variables. Cross-tabulation was used for categorical data. The Pearson Chi-square test was used to determine any statistically significant relationship between two variables in a crosstabulation. All crosstabulations had at least five expected observations per category. Results were considered statistically significant at the 5% significance level.

### 2.6 Open-ended question analysis

Open-ended survey question responses underwent qualitative data analysis using inductive thematic coding (30) in Excel. A student research assistant (MK) and the research assistant (BJ) reviewed the first five responses of the first open-ended question together to establish consistent coding. The student research assistant then coded the remaining responses. The research assistant reviewed this analysis fully at the endpoint to ensure consistent and reliable coding. In an effort to best interpret the respondents’ answers, given that neither MK nor BJ has lived experienced as an international student, they tried to think from the perspective of the international students they know and the international communities to which they belong. MK is a dental student of South Asian background and BJ is a second-generation immigrant and settler woman of South Asian and German descent. Both have experience with qualitative analysis from previous research projects.

### 2.7 Ethical considerations

The University of Saskatchewan Behavioural Research Ethics Board approved this study (Beh REB#5046). Participation was voluntary, and informed consent was obtained electronically for all participants via the first survey question.

## 3 Results

In total, *n* = 139 respondents consented to participate; *n* = 79 were currently enrolled as international students and eligible to participate. Of these *n* = 79 respondents, *n* = 56 answered at least one question about oral health and were included in the analyses. Percentages in the results and discussion sections are calculated using the number of responses to each question.

### 3.1 Demographics

Table 1 shows demographic survey data and key survey findings. The average age of respondents was 26.4 years (range 17–43 years).

**Table 1:** Summary of survey demographics, and key survey findings.

Variable	*n* (%)
Degree Pursued	
Undergraduate	23 (41.1)
Master's	18 (32.1)
Doctoral	15 (26.8)
Academic Unit	
Arts and Science	26 (46.4)
Health	13 (23.2)
Agriculture and Bioresources	5 (8.9)
Engineering	4 (7.1)
Graduate and Postdoctoral Studies	4 (7.1)
Other	4 (7.1)
Age (Mean)	26.4 years
Community Before Canada	
Urban	52 (94.5)
Rural	3 (5.5)
Sex	
Female	41 (73.2)
Male	15 (26.8)
Place of Residence Before Canada	
Same as Passport Nationality	44 (84.6)
Different Than Passport Nationality	8 (15.4)
Time Lived in Canada	
3 Months or Less	2 (3.6)
4–6 Months	4 (7.1)
7–12 Months	7 (12.5)
13–23 Months	16 (28.6)
24–35 Months	6 (10.7)
36 Months or More	21 (37.5)
Job Status	
Has a Job	33 (58.9)
Does Not Have a Job	23 (41.1)
Weekly Hours Worked at Job	
10 Hours or Less	8 (24.2)
11–20 Hours	16 (48.5)
21–30 Hours	7 (21.2)
31–39 Hours	2 (6.1)
Living Situation	
With a Roommate	22 (39.3)
Alone	18 (32.1)
With Their Family	11 (19.6)
With a Partner	7 (12.5)
With a Host Family	3 (5.4)
Food Security	
Enough to Eat AND Enough of Preferred Foods	23 (42.6)
Enough to Eat But NOT Enough of Preferred Foods	28 (51.9)
Not Enough to Eat	2 (3.7)
Source of Oral Health Information	
Oral Health Practitioners	32 (57.1)
Google	30 (53.6)
Parents	28 (50.0)
Previous Curriculum (e.g., elementary school, junior high, high school)	23 (41.1)
Social Media	15 (26.8)
Post-secondary School	5 (8.9)
Relatives/Friends	5 (8.9)
Oral Health Rating	
Poor	3 (5.4)
Fair	18 (32.1)
Good	25 (44.6)
Very Good	10 (17.9)
Excellent	0 (0.0)
Mental Health Rating	
Poor	7 (12.5)
Fair	15 (26.8)
Good	27 (48.2)
Very Good	2 (3.6)
Excellent	5 (8.9)
Overall Health Rating	
Poor	2 (3.6)
Fair	19 (33.9)
Good	25 (44.6)
Very Good	7 (12.5)
Excellent	3 (5.4)
Last Visit to Oral Health Professional	
Less Than 6 Months Ago	24 (42.9)
7–12 Months Ago	11 (19.6)
13 Months to Less Than 3 Years Ago	10 (17.9)
3 Years Ago or More	7 (12.5)
Never Seen an Oral Health Professional	4 (7.1)
Any Recent Mouth Pain	
Often	2 (3.6)
Sometimes	10 (17.9)
Rarely	18 (32.1)
Never	26 (46.4)

#### 3.1.1 Knowledge and attitudes

##### 3.1.1.1 Oral health knowledge and attitudes

**Figure 1:**
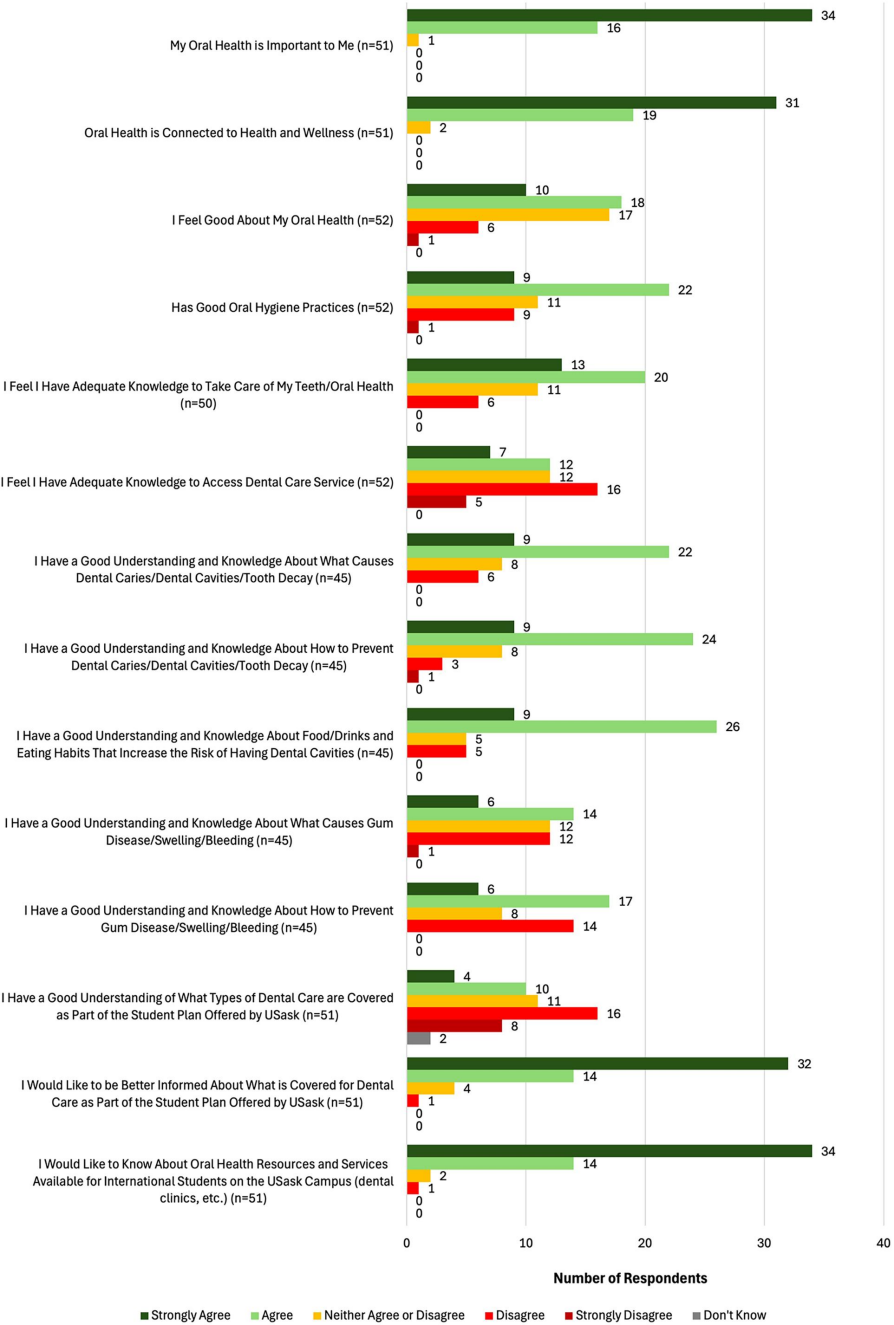
Knowledge and attitudes towards oral health.

Almost all (98.0%; *n* = 50) respondents “agree[d]” or “strongly agree[d]” that their oral health is important to them (see Figure 1). Fewer respondents (53.8%; *n* = 27) “agree[d]” or “strongly agree[d]” that they felt good about their oral health. In total, 40.4% (*n* = 21) of respondents “disagree[d]” or “strongly disagree[d]” that they had adequate knowledge to access dental care.

A vast majority (86.8%; *n* = 46) of respondents reported being familiar with the terms “dental caries,” “dental cavities,” and/or “tooth decay.” Fewer respondents “agree[d]” or “strongly agree[d]” that they had a “good understanding” of preventing these conditions (73.3%; *n* = 33). Students who reported “strongly agree[ing]” or “agree[ing]” that they knew what caused cavities were more likely to self-report having at least “good” oral health (74.2%; *n* = 23) compared to students who did not know about what caused cavities (28.6%; *n* = 4) (*p* < .05). Also, 40.0% (*n* = 22) of respondents reported having untreated mouth problems.

For students who had not visited an oral health practitioner in the last 12 months (37.5%; *n* = 21; see Supplementary Figure S1), the most common reasons were “no need for healthcare if no pain/problems” (66.7%; *n* = 14) and “financial constraints” (57.1%; *n* = 12).

##### 3.1.1.2 Diet knowledge and attitudes

Respondents (84.0%; *n* = 42) largely “agree[d]” or “strongly agreed” that they had good knowledge on the types of foods and drinks that were good for their overall health. Fewer respondents (66.7%; *n* = 36) “agree[d]” or “strongly agree[d]” that they had good knowledge on which foods and drinks were good for their oral health. Respondents who “strongly agree[d]” or “agree[d]” that they had good knowledge on food and drinks that were good for their oral health were more likely to report “good” or better oral health (69.4%; *n* = 25) compared to respondents who reported “neither disagree or agree,” “disagree,” or “strongly disagree” (41.2%; *n* = 7) (*p* < .05).

Respondents expressed inconsistent confidence in diet and nutrition. Forty-four percent (*n* = 24) of respondents “agree[d]” or “strongly agree[d]” that they had “good knowledge on the sugar content of foods and drinks in Canada.” As well, 77.8% (*n* = 35) respondents “agree[d]” or “strongly agree[d]” that they had a good understanding and knowledge about food/drinks and eating habits that increase the risk of having dental cavities.

#### 3.1.2 Oral health practices

##### 3.1.2.1 Accessing oral healthcare

Many respondents (62.5%; *n* = 35) visited an oral health professional within the past year. The most common reasons for seeing an oral health professional were routine checkups (55.8%; *n* = 29), preventative care (42.3%; *n* = 22), and treatments for dental conditions, including cavities or braces (36.5%; *n* = 19). However, 19.6% (*n* = 11) reported not seeing an oral health professional within the past three years or ever.

Twenty-five percent (*n* = 14) of respondents reported returning to their home country for oral care since beginning their studies at USask. Those who received oral health care in their home country since coming to Canada cited financial reasons (78.6%; *n* = 11), familiarity with the healthcare system (64.3%; *n* = 9), and more accessibility and/or comfort with the dental care provider (57.1%; *n* = 8). For a separate question, “Where did your most recent dental visit take place?”, most respondents (70.6%; *n* = 12) reported that their most recent dental visit was in their home country.

##### 3.1.2.2 Brushing

**Figure 2:**
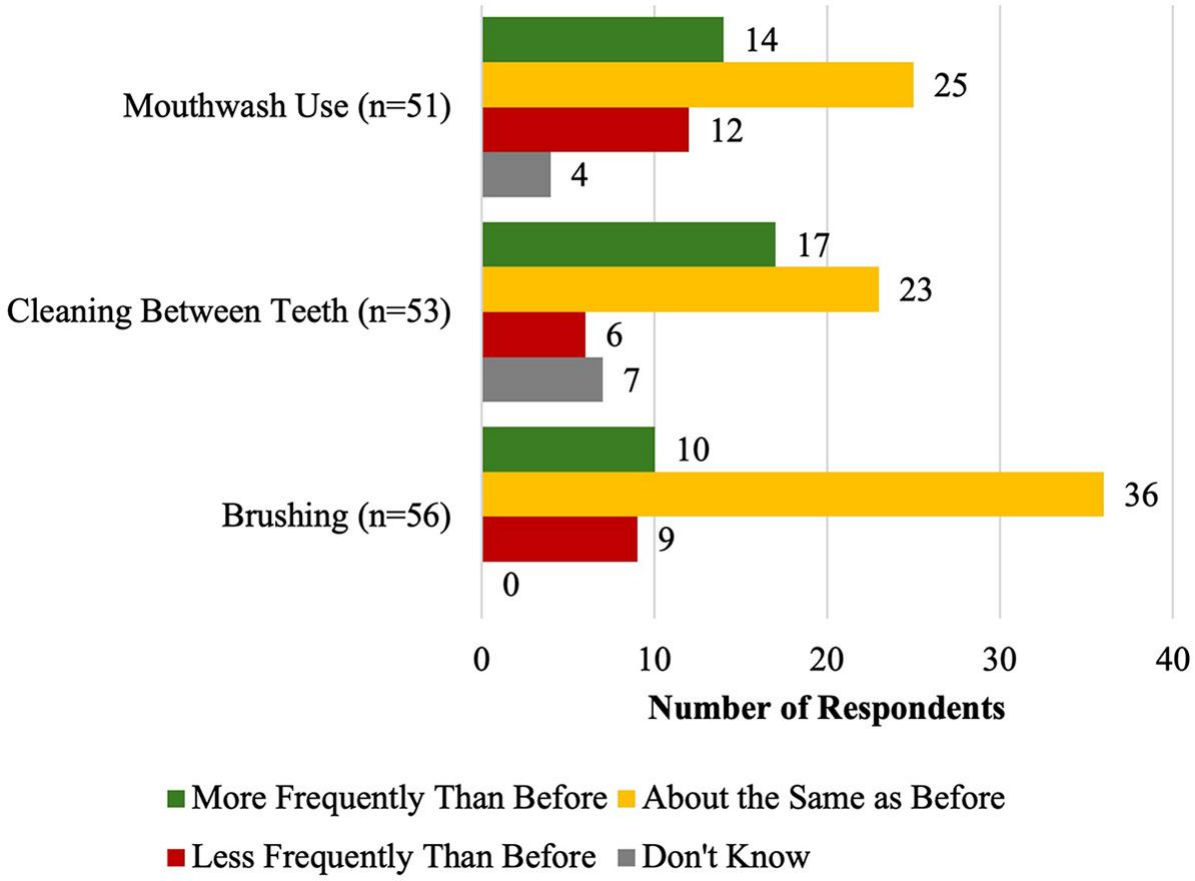
Oral health practice frequency since coming to Canada.

Many students kept the same oral practices for brushing (49.0%; *n* = 25), flossing (43.4%; *n* = 23), and mouthwash use (45.5%; *n* = 25) since coming to Canada (see Figure 2). Almost half of the respondents (42.9%; *n* = 24) reported brushing their teeth once daily. Over half of respondents (55.4%; *n* = 31) reported brushing their teeth twice daily and 21.8% (*n* = 12) of respondents reported using an electric toothbrush. There was no statistically significant relationship between frequency of brushing and self-reported oral health rating.

##### 3.1.2.3 Flossing

About one-third (36.4%; *n* = 20) of respondents reported never flossing and an additional 36.4% (*n* = 20) reported flossing twice per week or less often. Fewer respondents reported flossing three or four times (9.1%; *n* = 5), five or six times (7.3%; *n* = 4), or seven or more times (10.9%; *n* = 6) per week.

##### 3.1.2.4 Mouthwash use

Most (51.8%; *n* = 29) respondents never used mouthwash. Students who never used mouthwash were more likely to rate their oral health as “good,” “very good,” or “excellent” (75.9%; *n* = 22) than students who used mouthwash (44.0%; *n* = 11) (*p* < .05). Respondents who used mouthwash “more frequently than before” coming to Canada were less likely to rate their oral health as “good,” “very good,” or “excellent” (35.7%; *n* = 5) compared to respondents who used mouthwash “about the same” or “less frequently than before” coming to Canada (70.3%; *n* = 26) (*p* < .05).

##### 3.1.2.5 Dietary practices

**Figure 3:**
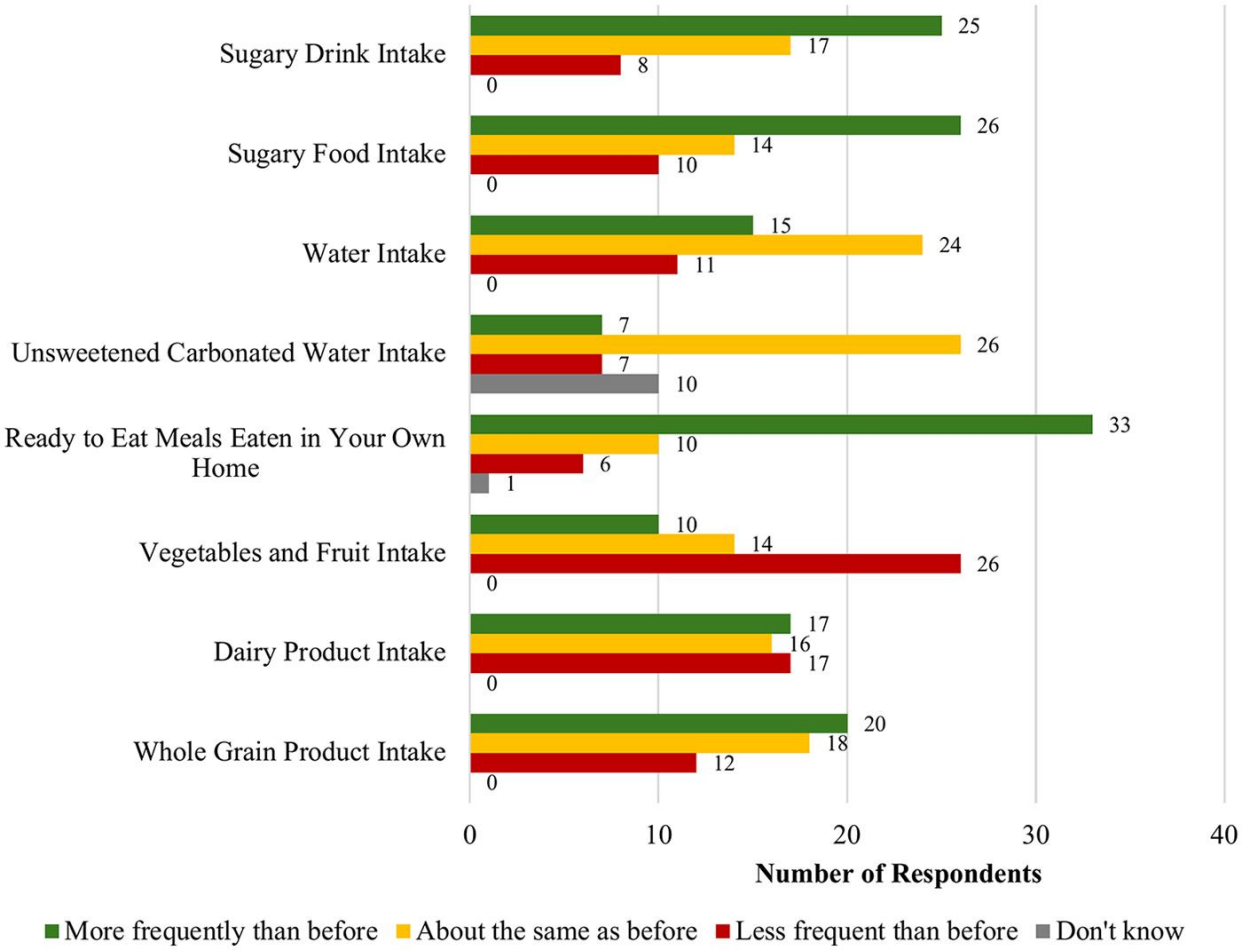
Dietary practices since coming to Canada (*n* = 50).

Overall, 31.4% (*n* = 17) of respondents “agree[d]” or “strongly agree[d]” that they ate a healthy diet daily in Canada. Respondents compared their current dietary practices to those practices before they relocated to Canada as international students (see Figure 3).

Two-thirds (66.0%; *n* = 33) reported eating more prepared at home “ready-to-eat meals” since arriving in Canada. Most respondents reported consuming more sugary foods (52.0%; *n* = 26) and sugary drinks (50.0%; *n* = 25) in Canada compared to when they were in their home country. On the other hand, 52.0% (*n* = 26) of respondents consumed fewer vegetables and fruits in Canada—the same number and percent as those who reported consuming more sugary foods.

##### 3.1.2.6 Respondent-identified gaps in knowledge, attitudes, and practices

Students were overwhelmingly interested in gaining oral health knowledge (see Supplementary Figure S2); all respondents selected at least one topic for further learning, including dental insurance coverage (84.3%; *n* = 43), followed by information about accessing oral healthcare in Canada (68.6%; *n* = 35), oral hygiene (64.7%; *n* = 33), and nutrition and oral health (60.7%; *n* = 31).

When asked about delivery of oral health education and awareness information, respondents preferred online written resources. Written resources on social media were the most popular (57.1%; *n* = 28), followed by online written resources—not social media (53.1%; *n* = 26), videos on social media (51.0%; *n* = 25), and promotional campaigns (44.9%; *n* = 22).

#### 3.1.3 Open-ended question responses

Respondents were also asked open-ended questions. When asked: “What do you feel is needed to improve the oral health of international students at USask?” two main themes emerged from a total of 35 respondents. First, respondents requested increased oral health education and awareness (62.9%; *n* = 22). A female respondent aged between 30 and 40 years old expressed that “[m]ore information upon arriving in Canada would be really helpful. I felt completely lost—I didn't know what was covered, and I have friends who were just as unsure.” Second, respondents wanted greater accessibility and affordability of oral healthcare (28.6%; *n* = 10), such as sourcing provider discounts and more comprehensive dental insurance coverage.

When asked: “What do you feel are the barriers/challenges regarding oral health for international students at USask?”, the most frequently mentioned barriers from a total of 40 respondents were difficulties navigating the oral healthcare system (52.5%; *n* = 21) and financial concerns (45.0%; *n* = 18). One respondent (a different female aged between 30 and 40 years old) cited a “lack of money and insurance coverage is small.” A different respondent (a male between 20 and 30 years old) explained he had “[i]nadequate information on how to access free/less costly services. Also, lack of information on how best to practice good oral health care.”

When asked: “What do you feel are the positives/opportunities regarding oral health for international students at USask?” 27 respondents provided answers. Overall, respondents liked the affordability of student insurance and the accessibility of various oral healthcare services—noting the insurance coverage (33.3%; *n* = 9), the dental clinic on campus (29.6%; *n* = 8), and opportunities for oral health education and promotion (18.5%; *n* = 5). Another respondent (a female participant aged between 20 and 30 years old) mentioned the insurance coverage and suggested educational interventions with potential financial positives:

“The university offers a student dental plan that can help cover some costs for preventive and basic dental treatments. Opportunities exist to educate students on oral hygiene, insurance usage, and available services. Multicultural student organizations or health initiatives could help spread awareness and provide support. Access to information and early prevention measures can reduce future dental costs.”

When asked: “In the past 12 months, which foods have you avoided eating because of problems with your mouth?” (*n* = 25), respondents indicated they avoided consuming sugary foods and/or drinks (28.0%; *n* = 7), hard/chewy foods (24.0%; *n* = 6), and hot and cold foods and drinks (20.0%; *n* = 5).

## 4 Discussion

This study aimed to explore self-reported knowledge, attitudes, and practices regarding oral health and oral health preventative and therapeutic practices of international students at a large Canadian university. To our knowledge, this is the first Canadian study of its kind.

Students value oral healthcare but could benefit from more knowledge on navigating Canadian oral healthcare (including insurance), dental hygiene, and diet to improve their oral health. This finding echoes research from other countries, which revealed limited understanding about oral health (7, 14, 16, 31). As well, some studies have reported that international students tend to consume less healthy foods and beverages since coming to Canada (32, 33). Moreover, these students may not have access to their preferred foods. This lack of access to preferred foods may be their cultural foods, as Hanbazaza et al. (34) found. Our study findings reveal that international students largely want more information about dental insurance, how to access oral healthcare in Canada, and dental hygiene, and potentially diet and nutrition, mainly through written, online resources. This aligns with diet and nutrition research on international students in Canada that recommends developing resources to help them find healthy foods and build healthy eating habits (32–34). Overall, the demand for resources to help international students is mentioned in other articles (7, 14, 31, 34) and was also found in this study.

Following Yassin et al.'s (7) scoping review on international students’ oral health in Canada, students who completed this survey indicate that website content and/or a social media page on dental insurance and other aspects of oral health would benefit them. Conducting focus groups to both further examine these suggestions and an implementation strategy for potential interventions (including involvement of student organizations) would provide a path forward for sharing oral health knowledge with international students.

A comparison with Statistics Canada (27) data for Saskatchewan residents aged 18–34 years (most similar demographic to survey respondents) indicates potentially worse oral health for international students for every statistic (see Table 2). Although this data for Saskatchewan residents aged 18–34 provides the closest available comparison, it would include a wide variety of individuals including those who are domestic students, individuals who are employed full-time or part-time, or are unemployed. Beyond work or student status, these residents of Saskatchewan may have one of many migration statuses. In regards to dental care coverage, Saskatchewan residents have varied insurance coverage status, whereas students (international and domestic) at USask are required to enroll in dental insurance upon registration. Compared to international students at USask, Saskatchewan residents between 18 and 34 years old may be more accustomed to navigating the Canadian oral healthcare system, including dental insurance compared to new-to-Canada international students. Accordingly, there are nuanced differences in individual circumstances and the resources available to these groups may have some overlap. Our international student data suggests that there are some potential oral health concerns in this group. It shows a need for future in-depth research to better understand these potential issues. Future studies should specifically conduct comprehensive clinical exams to understand the oral disease burden and compare international students' oral health status with other groups.

**Table 2:** Comparison of our survey findings with Statistics Canada.

Oral Health Attitude/Practice	% Respondents	
	Statistics Canada (18–34 year olds)	Our Survey (International Students)
Avoid eating foods because of mouth problems, sometimes or often	16.8%	23.2%
Brush teeth 2 or more times per day	65.7%	55.4%
Floss 5 or more times per week	26.6%	18.2%
Perceive oral health as “fair” or “poor”	11.8%	32.1%
Perceive oral health as “very good” or “excellent”	55.7%	17.9%
Experience persistent or on-going mouth pain, sometimes or often	18.6%	21.4%

All Statistics Canada data in Table 2 is from the Canadian Oral Health Survey (35).

Approximately one million international students are studying in Canada (6), many of whom become permanent residents and citizens (26). Since undergraduate and doctoral students require four or more years for degree completion, they spend substantial time in Canada. Since international students at USask are required to have dental insurance, it is notable that 70.6% (*n* = 12) of international students reported returning to their home country to visit an oral healthcare professional. Further, it is concerning that nearly half (40.0%; *n* = 22) of respondents report having untreated mouth problems. Some combination of financial barriers, a lack of clarity around how to access this insurance, and other factors appear to result in international students not seeking oral healthcare in Canada, even if they have untreated mouth problems. Providing resources that help international students understand their insurance coverage and how to access oral care in Canada is key to improving their oral health.

This study has strengths and limitations. A key strength of this study is that it provides baseline data on the oral health of international students in Canada, while it is specific to one university in Saskatchewan. Further, feedback on the study design and survey questionnaire from student organizations and individual USask international students maximized the accessibility, reach, and clarity of the survey. One limitation is that while researchers recruited survey respondents using the various approved channels, the resulting sample size was relatively small. Due to the small sample size, caution should be applied when interpreting the findings from the subgroup analyses and chi-square tests. Additionally, this survey may have attracted respondents who are more interested in oral health compared to the general international student population.

## 5 Conclusion

The results from our study found that many international students reported limited oral health knowledge, changes in their diets since coming to Canada, and poor oral care practices. International students reported various indicators of poor oral health. Strategies to reduce the disparities were suggested by participants, but more detailed investigation is necessary to design appropriate interventions. These findings suggest that universities, student health services, and policymakers in student support organizations could work together to develop and provide culturally appropriate insurance orientation sessions and online written information to international students. Co-creating interventions with international students and organizations ensures that the delivery method and resources on oral healthcare in Canada match international students’ needs. Also, the process of co-creation could build the confidence of participating international students. Given the relatively small sample size, future research could expand the study to universities across Canada and enable researchers to get a larger representative sample. Finally, objective data from clinical oral health examinations could help to substantiate self-reported data and gain a clearer picture of the clinical oral health status of the international student population. These methods would respectively allow for greater insight into international students’ oral health, which is needed to design and test future interventions.

## Data Availability

The datasets presented in this article are not readily available because of confidentiality and ethical reasons since it could identify participants through the triangulation of their responses. Requests to access the datasets should be directed to amrinderbir.singh@usask.ca.

## Supplementary material

Supplementary Table S1 Key variables.

Supplementary Table S2 Key dependent variables.

Supplementary Figure S1 Reasons for not visiting an oral health professional (*n* = 21).

Supplementary Figure S2 Oral health topics international students want to learn about (*n* = 51).
