# Supplementary material for: Surveying the oral health needs of international students in Canada
Source: Front Oral Health. 2026 Apr 22;7:1766729. doi: 10.3389/froh.2026.1766729 (PMC13144017; doi:10.3389/froh.2026.1766729)
Supplement: Supplementary Table S1 — Key variables. [file Table1.docx]

Supplementary Material

# Supplementary Table 1. Key Variables.

| **Concept** | **Indicator** | **Variable Name** | **Observations** | **Median** | **IQR** | **Mode** | **Min, Max** | **Measure** |
| --- | --- | --- | --- | --- | --- | --- | --- | --- |
| **Degree Pursued** | What level of education are you currently pursuing at USask? | degree | 71 | - | - | 1 | (1, 3) | 1=Undergraduate 2=Master's 3=Doctoral |
| **Academic Unit** | Please indicate your current USask academic unit (college/school). | acadunit | 71 | - | - | 2 | (1, 18) | 1=Agriculture and Bioresources 2=Arts and Science 3=Dentistry 4=Education 5=Edwards School of Business 6=Engineering 7=School of Environment and Sustainability 8=Graduate and Postdoctoral Studies 9=Kinesiology 10=Law 11=Library 12=Medicine 13=Nursing 14=Pharmacy and Nutrition 15=School of Rehabilitation Science 16=School of Public Health 17=Johnson-Shoyama Graduate School of Public Policy 18=Veterinary Medicine 19=Briercrest College 20=St. Thomas More College 21=Horizon College & Seminary 22=Gabriel Dumont Institute of Native Studies & Applied Research 23=St. Peter's College 24=The College of Emmanuel and St. Chad 25=St. Andrew’s College 26=Lutheran Theological Seminary |
| **Age** | What is your age in years? | age_buckets | 58 | - | - | 2 | (1, 2) | 1=17–24 years old 2=25–64 years old |
| **Community** | Prior to coming to USask, what type of community best describes where you spent the most amount of time of your life? | urbanrural | 51 | - | - | 1 | (1, 2) | 1=Urban (metropolitan area, i.e., city with all services) 2=Rural (areas outside metropolitan areas, i.e., towns, villages with basic services and those living on farms near cities, towns, or villages) 3=Remote (communities located very far from a cities, towns, or villages) |
| **Sex** | What is your biological sex? | sex | 61 | - | - | 2 | (1, 2) | 1=Male 2=Female |
| **Time Lived in Canada** | How long have you been residing in Canada? | timeincda | 70 | 2.00 | 2.00 | - | (1, 4) | 1=12 months or less 2=13-23 months 3=24-35 months 4=36 months or more |
