# Supplementary material for: Surveying the oral health needs of international students in Canada
Source: Front Oral Health. 2026 Apr 22;7:1766729. doi: 10.3389/froh.2026.1766729 (PMC13144017; doi:10.3389/froh.2026.1766729)
Supplement: Supplementary Table S2 — Key dependent variables. [file Table2.docx]

Supplementary Material

# Supplementary Table 2. Key Dependent Variables

| **Concept** | **Indicator** | **Variable Name** | **Observations** | **Median** | **IQR** | **Mode** | **Min, Max** | **Measure** |
| --- | --- | --- | --- | --- | --- | --- | --- | --- |
| **Source of Oral Health Knowledge** | Where does most of your oral health knowledge come from? Please select all that apply. [Previous curriculum in school (e.g., elementary, junior high, high school)] | prevschool | 56 | - | - | 2 | (1, 2) | 1=Not Selected 2=Selected |
|  | Where does most of your oral health knowledge come from? Please select all that apply. [Curriculum in university/college] | postsecondaryschool | 56 | - | - | 1 | (1, 2) |  |
|  | Where does most of your oral health knowledge come from? Please select all that apply. [Parents] | parents | 56 | - | - | 2 | (1, 2) |  |
|  | Where does most of your oral health knowledge come from? Please select all that apply. [Relatives/Friends] | relativesfriends | 56 | - | - | 1 | (1, 2) |  |
|  | Where does most of your oral health knowledge come from? Please select all that apply. [Oral health practitioners (dentist, dental hygienist, dental therapists, etc.)] | practitioners | 56 | - | - | 2 | (1, 2) |  |
|  | Where does most of your oral health knowledge come from? Please select all that apply. ["Google"] | google | 56 | - | - | 2 | (1, 2) |  |
|  | Where does most of your oral health knowledge come from? Please select all that apply. [Social Media] | sourcesocialmedia | 56 | - | - | 1 | (1, 2) |  |
|  | Where does most of your oral health knowledge come from? Please select all that apply. ["I have never received any dental education"] | nodentedu | 56 | - | - | 1 | (1, 2) |  |
| **Knowledge and Attitudes Towards Oral Health** | My oral health is important to me | oralhealthimp | 51 | 5.00 | 1.00 | - | (3, 5) | 1=Strongly Disagree 2=Disagree 3=Neither Disagree or Agree 4=Agree 5=Strongly Agree |
|  | Oral health is connected to health and wellness | healthconnex | 56 | 5.00 | 1.00 | - | (2, 5) |  |
|  | Currently, I feel good about my oral health | oralhealthgood | 52 | 4.00 | 1.00 | - | (1, 5) |  |
|  | I feel that I have a good routine for my oral health hygiene practices (e.g. brushing, flossing, mouthwash use, etc.) | oralhealthpractices | 55 | 4.00 | 1.00 | - | (1, 5) |  |
|  | Moving to Canada has positively impacted my oral health | cdnoralhealth | 55 | 3.00 | 2.00 | - | (1, 5) |  |
|  | Moving to Canada has positively impacted my overall health | cdngenhealth | 56 | 3.00 | 2.00 | - | (1, 5) |  |
|  | I feel that I have adequate knowledge to take care of my teeth/oral health | adqinfohealth | 52 | 4.00 | 3.00 | - | (2, 5) |  |
|  | I feel that I have adequate knowledge on how to access dental care services in Canada | adqinfodntsrv | 55 | 3.00 | 2.00 | - | (1, 5) |  |
|  | I have a good understanding and knowledge about what causes dental caries/dental cavities/tooth decay | knowcausecavity | 45 | 4.00 | 4.00 | - | (1, 5) | 1=Strongly Disagree  2=Disagree  3=Neither Disagree or Agree  4=Agree  5=Strongly Agree  6=Don't Know |
|  | I have a good understanding and knowledge about how to prevent dental caries/dental cavities/tooth decay | knowpreventcavity | 45 | 4.00 | 4.00 | - | (1, 5) |  |
|  | I have a good understanding and knowledge about what causes gum disease/swelling/bleeding | knowcausebleed | 45 | 3.00 | 4.00 | - | (1, 5) |  |
|  | I have a good understanding and knowledge about how to prevent gum disease/swelling/bleeding. | knowpreventbleed | 45 | 4.00 | 3.00 | - | (2, 5) |  |
|  | I have a good understanding of what types of dental care are covered as part of the student plan offered by USask | understandusaskplan | 51 | 3.00 | 5.00 | - | (1, 6) |  |
|  | I would like to be better informed about what is covered for dental care as part of the student plan offered by USask | moreinfousaskplan | 51 | 5.00 | 3.00 | - | (2, 5) |  |
|  | I would like to know about oral health resources and services available for international students on the USask campus (i.e. dental clinics, etc.) | moreinfousaskres | 51 | 5.00 | 3.00 | - | (2, 5) |  |
| **Health Rating** | In general, how would you rate your oral health? | rateoralhealth | 56 | 3.00 | 1.00 | - | (1, 4) | 1=Poor 2=Fair 3=Good 4=Very Good 5=Excellent |
|  | In general, how would you rate your overall health? | rategenhealth | 56 | 3.00 | 4.00 | - | (1, 5) |  |
|  | In general, how would you rate your mental health? | ratemtlhealth | 56 | 3.00 | 4.00 | - | (1, 5) |  |
| **Diet and Nutrition Knowledge and Attitudes** | The foods and drinks that I consume have an impact on my oral health. | fdimporal | 54 | 4.00 | 5.00 | - | (1, 6) | 1=Strongly Disagree  2=Disagree  3=Neither Disagree or Agree  4=Agree  5=Strongly Agree  6=Don't Know |
|  | The foods and drinks that I consume have an impact on my overall health. | fdimpgen | 54 | 5.00 | 3.00 | - | (2, 5) |  |
|  | I have good knowledge on the types of foods and drinks that are good for my oral health. | fdknoworal | 54 | 4.00 | 5.00 | - | (2, 6) |  |
|  | I have good knowledge on the types of foods and drinks that are good for my overall health. | fdknowgen | 54 | 4.00 | 3.00 | - | (2, 5) |  |
|  | I have good knowledge on the sugar content of foods and drinks in Canada. | fdknowsugar | 54 | 3.00 | 5.00 | - | (1, 6) |  |
| **Last Seen Oral Health Professional** | When was the last time that you saw an oral health professional (e.g., dentist, dental hygienist, dental therapist, or any other dental specialist)? | lastseenpro | 56 | 2.00 | 2.00 | - | (1, 5) | 1=Less than 6 months ago 2=7-12 months ago 3=13 months to less than 3 years ago 4=3 years ago or more 5=Never seen an oral health professional |
| **Reason for Not Seeing An Oral Health Specialist Recently** | What was the reason for not visiting an oral health professional within the last year? Please select all that apply. [Academic stress] | novisitstress | 21 | - | - | 1 | (1, 2) | 1=Not Selected 2=Selected |
|  | What was the reason for not visiting an oral health professional within the last year? Please select all that apply. [No need for oral health care if I do not feel any pain or am not experiencing any problems] | novisitnoprob | 21 | - | - | 2 | (1, 2) |  |
|  | What was the reason for not visiting an oral health professional within the last year? Please select all that apply. [ I don't know how to access an oral health professional] | novisitaccess | 21 | - | - | 1 | (1, 2) |  |
|  | What was the reason for not visiting an oral health professional within the last year? Please select all that apply. [Fear or anxiety related to dental visits] | novisitanxiety | 21 | - | - | 1 | (1, 2) |  |
|  | What was the reason for not visiting an oral health professional within the last year? Please select all that apply. [Financial constraints (expensive)] | novisitexpensive | 21 | - | - | 2 | (1, 2) |  |
|  | What was the reason for not visiting an oral health professional within the last year? Please select all that apply. [Lack of insurance coverage] | novisitlackcoverage | 21 | - | - | 1 | (1, 2) |  |
|  | What was the reason for not visiting an oral health professional within the last year? Please select all that apply. [I am not aware of Canada's oral health care system] | novisitcdasystem | 21 | - | - | 1 | (1, 2) |  |
|  | What was the reason for not visiting an oral health professional within the last year? Please select all that apply. [I do not understand how insurance coverage works] | novisitinsuranceconfusing | 21 | - | - | 1 | (1, 2) |  |
|  | What was the reason for not visiting an oral health professional within the last year? Please select all that apply. [Other] | novisitculture novisitscheduling novisitwaitlist novisitother | 21 | - | - | 1 | (1, 2) |  |
| **Location of Most Recent Dental Visit** | Where did your most recent dental visit take place? | lastdentvisit | 17 | - | - | 3 | (1, 3) | 0=In another country, please list:  1=Canada  2=USA  3=In my home country |
| **Oral Care in Home Country** | Have you returned to your home country and received any oral healthcare treatments in your home country since arriving in Canada to study at USask? | returnhomeoral | 56 | - | - | 2 | (1, 2) | 1=Yes  2=No |
|  | If you have received oral healthcare treatments in your home country since arriving in Canada to study at USask, what were the reasons? Please select all that apply. | returnaffordability  returnaccessibility  returncomfort  returnculturalsafety  returnfamiliarity | 13 | - | - | 1 | (1, 2) | 1=Not Selected 2=Selected |
| **Barriers to Oral Health** | What do you feel are the barriers/challenges regarding oral health for international students at USask? | barriers | 40 | - | - | - | - | Open-ended |
| **Positives/**  **Opportunities for Oral Health** | What do you feel are the positives/opportunities regarding oral health for international students at USask? | positives | 27 | - | - | - | - |  |
| **Changes Needed for Improved Oral Health** | What do you feel is needed to improve the oral health of international students at USask? | improve | 35 | - | - | - | - |  |
| **Oral Health Topics International Students Want to Learn About** | What types of topic areas would you like to learn more about to improve your oral health? [Oral hygiene (brushing, flossing, etc.)] | learnmorehygiene | 51 | - | - | 2 | (1, 2) | 1=Not Selected 2=Selected |
|  | What types of topic areas would you like to learn more about to improve your oral health? [Nutrition and oral health] | learnmorehealth | 51 | - | - | 2 | (1, 2) |  |
|  | What types of topic areas would you like to learn more about to improve your oral health? [Accessing healthy foods in Saskatoon] | learnmorefoodsask | 51 | - | - | 1 | (1, 2) |  |
|  | What types of topic areas would you like to learn more about to improve your oral health? [Accessing healthy foods on campus] | learnmorefoodcampus | 51 | - | - | 1 | (1, 2) |  |
|  | What types of topic areas would you like to learn more about to improve your oral health? [Tobacco and alcohol use and oral health] | learnmoresubstanceuse | 51 | - | - | 1 | (1, 2) |  |
|  | What types of topic areas would you like to learn more about to improve your oral health? [How to access oral health care in Canada] | learnmoreaccess | 51 | - | - | 2 | (1, 2) |  |
|  | What types of topic areas would you like to learn more about to improve your oral health? [Dental insurance coverage] | learnmoreinsurance | 51 | - | - | 2 | (1, 2) |  |
|  | What types of topic areas would you like to learn more about to improve your oral health? [Oral diseases (e.g., tooth decay/cavities, gum disease)] | learnmoredisease | 51 | - | - | 1 | (1, 2) |  |
| **Receive Information** | How would you like to receive this information? [Presentations/Information Sessions] | getpres | 49 | - | - | 1 | (1, 2) | 1=Not Selected 2=Selected |
|  | How would you like to receive this information? [Promotional campaigns] | getpromo | 49 | - | - | 1 | (1, 2) |  |
|  | How would you like to receive this information? [Written resources - printed] | getwrittenprint | 49 | - | - | 1 | (1, 2) |  |
|  | How would you like to receive this information? [Written resources - website] | getwrittenweb | 49 | - | - | 2 | (1, 2) |  |
|  | How would you like to receive this information? [Written resources - social media] | getwrittensm | 49 | - | - | 2 | (1, 2) |  |
|  | How would you like to receive this information? [Videos - social media] | getvidsm | 49 | - | - | 2 | (1, 2) |  |
|  | How would you like to receive this information? [Videos - website] | getvidweb | 49 | - | - | 1 | (1, 2) |  |
| **Dietary Practices Since Coming to Canada** | Since coming to Canada, I eat a healthy diet on a daily basis. | dietcda | 54 | 3.00 | 2.00 | - | (1, 5) | 1=Strongly Disagree 2=Disagree 3=Neither Disagree or Agree 4=Agree 5=Strongly Agree 6=Don't Know |
|  | Since coming to Canada, I consume more sugary foods and/or drinks than before I arrived in Canada. | sugarcda | 54 | 4.00 | 5.00 | - | (1, 6) |  |
|  | Since coming to Canada, I can access foods from my home country/culture. | homefoodcda | 54 | 3.00 | 5.00 | - | (1, 6) |  |
|  | Since moving to Canada, how have the following dietary practices changed for you? [Sugary drink intake (e.g., juice, soft drinks, sugary coffee drinks)] | sugarydrink | 54 | 2.00 | 2.00 | - | (1, 3) | 1=Less frequent than before 2=About the same as before 3=More frequently than before 4=Don't know |
|  | Since moving to Canada, how have the following dietary practices changed for you? [Sugary food intake (e.g., candy, pastries, donuts, cookies)] | sugaryfood | 54 | 2.00 | 1.00 | - | (1, 3) |  |
|  | Since moving to Canada, how have the following dietary practices changed for you? [Water intake (tap or bottled - non carbonated, still)] | water | 54 | 2.00 | 1.00 | - | (1, 3) |  |
|  | Since moving to Canada, how have the following dietary practices changed for you? ['Unsweetened carbonated water intake (e.g., flavoured or unflavoured - bubly, Perrier)] | sparklingwater | 54 | 2.00 | 1.00 | - | (1, 4) |  |
|  | Since moving to Canada, how have the following dietary practices changed for you? [Ready to eat meals eaten in your home (e.g., frozen or pre-made meals that you buy, heat, and serve, meals purchased from a grocery store kitchen/deli to eat at home)] | readytoeat | 54 | 3.00 | 1.00 | - | (1, 4) |  |
|  | Since moving to Canada, how have the following dietary practices changed for you? [Vegetables and fruit intake (not including fruit juice)] | fruitveg | 54 | 1.00 | 1.00 | - | (1, 3) |  |
|  | Since moving to Canada, how have the following dietary practices changed for you? [Dairy product intake (e.g., milk, yogurt, cheese)] | dairy | 54 | 2.00 | 2.00 | - | (1, 3) |  |
|  | Since moving to Canada, how have the following dietary practices changed for you? [Whole grain product intake (e.g., whole grain breads, bagels, wraps, crackers)] | grain | 54 | 2.00 | 1.00 | - | (1, 3) |  |
| **Oral Healthcare Practice Changes Since Coming to Canada** | Since moving to Canada, how has the frequency of the following oral healthcare practices changed for you? [Brushing] | brushingcda | 56 | 2.00 | 0.00 | - | (1, 4) | 1=Less frequent than before 2=About the same as before 3=More frequently than before 4=Don't know |
|  | Since moving to Canada, how has the frequency of the following oral healthcare practices changed for you? [Cleaning between teeth] | flossingcda | 53 | 2.00 | 1.00 | - | (1, 4) |  |
|  | Since moving to Canada, how has the frequency of the following oral healthcare practices changed for you? [Cleaning between teeth] | mouthwashcda | 51 | 2.00 | 1.00 | - | (1, 3) |  |
| **Oral Healthcare in Canada** | What are your current teeth brushing habits? | brushingfreq | 56 | 4.00 | 3.00 | - | (2, 5) | 1=Never  2=Less than once per day  3=Once per day  4=Twice per day  5=Three or more times per day |
|  | Currently, do you use an electric toothbrush? | Electrictoothbrush | 55 | - | - | 2.00 | (1, 2) | 1=Yes  2=No |
|  | Currently, how often do you usually clean between your teeth with dental floss or any flossing aid? | flossing | 55 | 2.00 | 5.00 | - | (1, 6) | 1=Never  2=Less than once per week  3=One or two times per week  4=Three or four times per week  5=Five or six times per week  6=Seven or more times per week  7=Don’t know |
|  | Currently, how often do you usually use mouthwash? | mouthwash | 56 | 1.00 | 5.00 | - | (1, 6) | 1=Never  2=Less than once per day  3=Once per day  4=Twice per day  5=Three or more times per day  6=Don't know |
| **Avoidance** | In the past 12 months, which foods have you avoided eating because of problems with your mouth? | avoid | 25 | - | - | - | - | Open-ended |
| **Returned Home for Oral Healthcare** | Have you returned to your home country and received any oral healthcare treatments in your home country since arriving in Canada to study at USask? | returnhomeoral | 17 | 2.00 | 0.75 | - | (1, 2) | 1=Yes 2=No |
|  | If you have received oral healthcare treatments in your home country since arriving in Canada to study at USask, what were the reasons? Please select all that apply. [Financial reasons (more affordable)] | returnaffordability | 14 | - | - | 2 | (1, 2) | 1=Not Selected 2=Selected |
|  | If you have received oral healthcare treatments in your home country since arriving in Canada to study at USask, what were the reasons? Please select all that apply. [More accessible (shorter waiting times, more dentists are available)] | returnaccessibility | 14 | - | - | 2 | (1, 2) |  |
|  | If you have received oral healthcare treatments in your home country since arriving in Canada to study at USask, what were the reasons? Please select all that apply. [Comfort with the dental care provider] | returncomfort | 14 | - | - | 2 | (1, 2) |  |
|  | If you have received oral healthcare treatments in your home country since arriving in Canada to study at USask, what were the reasons? Please select all that apply. [Felt culturally safe] | returnculturalsafety | 14 | - | - | 1 | (1, 2) |  |
|  | If you have received oral healthcare treatments in your home country since arriving in Canada to study at USask, what were the reasons? Please select all that apply. [Familiarity with the healthcare system] | returnfamiliarity | 14 | - | - | 2 | (1, 2) |  |
| **Oral Health Care Practices** | What are your current teeth brushing habits? | brushing | 56 | 4.00 | 1.00 | - | (2, 5) | 1=Never 2=Less than once per day 3=Once per day 4=Twice per day 5=Three or more times per day |
|  | Currently, how often do you usually clean between your teeth with dental floss or any flossing aid? | flossing | 55 | 2.00 | 3.00 | - | (1, 6) | 1=Never 2=Less than once per week 3=One or two times per week 4=Three or four times per week 5=Five or six times per week 6=Seven or more times per week 7=Don't know |
|  | Currently, how often do you usually use mouthwash? | mouthwash | 56 | 1.00 | 1.75 | - | (1, 6) | 1=Never 2=Less than once per day 3=Once per day 4=Twice per day 5=Three or more times per day 6=Don't know |
