# Supplementary figures and images for: Surveying the oral health needs of international students in Canada
Source: Front Oral Health. 2026 Apr 22;7:1766729. doi: 10.3389/froh.2026.1766729 (PMC13144017; doi:10.3389/froh.2026.1766729)

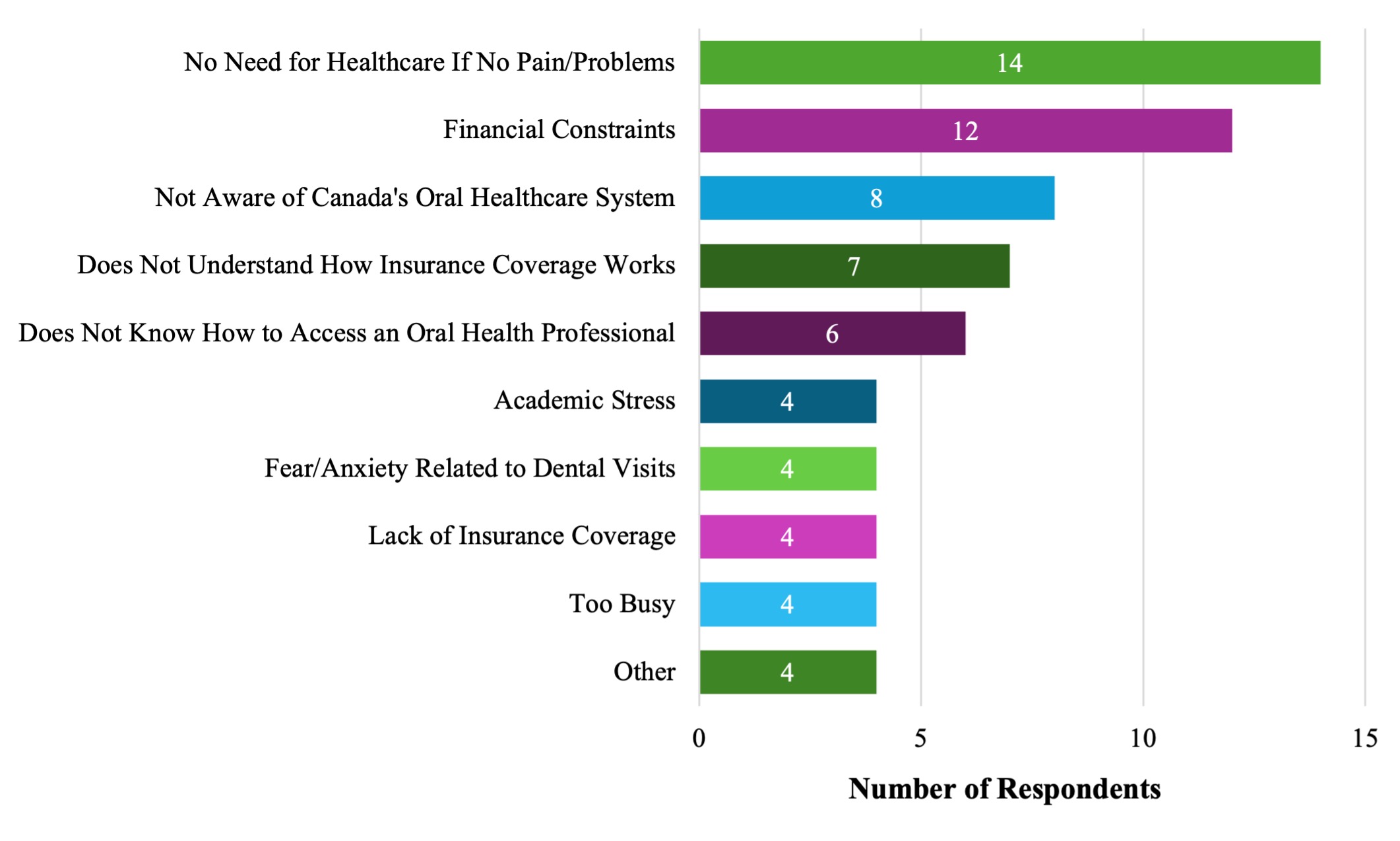

Supplement: Supplementary Figure S1 — Reasons for not visiting an oral health professional (n = 21). [file Image1.jpg]

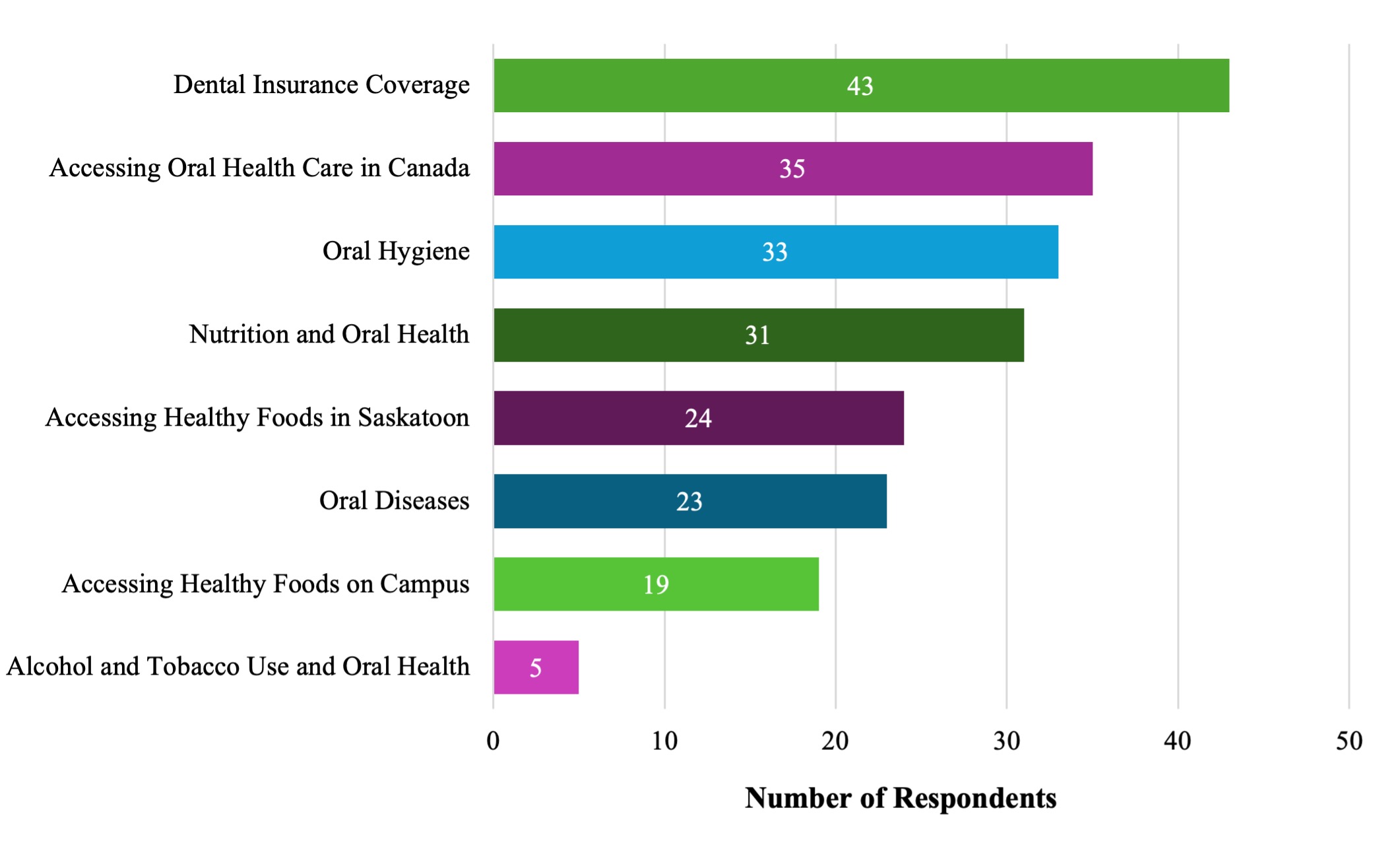

Supplement: Supplementary Figure S2 — Oral health topics international students want to learn about (n = 51). [file Image2.jpg]
